# Effects of dexmedetomidine and magnesium sulphate on controlled hypotension in FES surgeries

**DOI:** 10.6026/973206300211211

**Published:** 2025-05-31

**Authors:** Rajni Thakur, Shashi Kumari, Kirti Ahirwal, Sivasamy S.

**Affiliations:** 1 Department of Anaesthesia, Gandhi medical College, Bhopal, Madhya Pradesh, India

**Keywords:** Dexmedetomidine, magnesium sulphate, controlled hypotension, endoscopic sinus surgery, functional endoscopic sinus surgery (FESS)

## Abstract

Rhinosinusitis often requires functional endoscopic sinus surgery (FESS) when medical management fails, and controlled hypotension minimizes blood loss and optimizes surgical conditions. Dexmedetomidine and magnesium sulphate are commonly used to achieve controlled hypotension. Therefore, it is of interest to compare their efficacy in FESS, randomizing 60 patients to receive either dexmedetomidine or magnesium sulphate. Intraoperative mean arterial pressure (MAP) was consistently lower in the dexmedetomidine group compared to the magnesium sulphate group (p<0.01). We show that dexmedetomidine demonstrated superior performance for controlled hypotension during FESS, potentially improving surgical conditions and patient outcomes.

## Background:

Rhinosinusitis is a common clinical condition affecting individuals of all ages and genders. For patients with persistent sinusitis that does not respond to medical therapy, endoscopic sinus surgery is often recommended as an effective treatment. However, due to the location of the surgery, even small amounts of blood can obscure the surgeon's vision, making the procedure more difficult and time-consuming. This can lead to complications such as cerebrospinal fluid leaks, intracranial infections, orbital issues, significant bleeding and the need for blood transfusions or surgical intervention [1- 2]. The nasal cavity, rich in blood vessels, helps to moisten and warm inspired air, making it prone to significant bleeding during surgery. Even minor bleeding can obstruct vision, increase the risk of complications, extend the surgery duration and diminish the quality of the procedure. In endoscopic sinus surgeries, where success is heavily reliant on a clear surgical field, bleeding is a major factor that can hinder the process. To manage this, various techniques such as using local vasoconstrictors, controlling posture, pharmacological cardio-depression and preoperative steroids are employed to reduce bleeding [1]. One important technique is "controlled hypotension," which involves gradually lowering the arterial blood pressure to minimize surgical blood loss, improve vision and reduce complications. For an ideal agent to be used in controlled hypotension, it must have certain characteristics: easy administration, a brief onset period, rapid elimination without harmful metabolites, minimal impact on vital organs and predictable, dose-dependent effects [4]. Several agents, including inhalational agents like halothane, intravenous propofol, vasodilators such as sodium nitroprusside, beta blockers, alpha-adrenergic agonists and other agents like remifentanil and magnesium sulphate, are commonly used for this purpose [2-3]. Beta-blockers, in particular, help reduce local bleeding by lowering cardiac output without causing significant systemic vasodilation. On the other hand, sodium nitroprusside, a vasodilator, may increase local bleeding due to vasodilation and reflex tachycardia. Dexmedetomidine, a selective α2-receptor agonist, is another useful agent for controlled hypotension, offering analgesic, anxiolytic and sympatholytic properties, which are beneficial in anesthesia. It works by reducing heart rate, arterial blood pressure and norepinephrine release [5, 6-7]. Magnesium sulphate is another promising agent for controlled hypotension, as it helps stabilize cell membranes and intracytoplasmic organelles. It activates enzymes involved in transmembrane ion exchange during depolarization and repolarization. Additionally, magnesium inhibits the release of norepinephrine and reduces blood pressure by blocking N-type calcium channels at nerve endings, further contributing to its effectiveness in controlled hypotension [8]. Therefore, it is of interest to report the effects of dexmedetomidine and magnesium sulphate on controlled hypotension in FES surgeries.

## Hypothesis:

## Null hypothesis:

There is no significant difference in the effectiveness of dexmedetomidine and magnesium sulphate in achieving controlled hypotension during FESS surgeries.

## Alternative hypothesis:

There is a significant difference in the effectiveness of dexmedetomidine and magnesium sulphate in achieving controlled hypotension during FESS surgeries.

## Aims and Objectives:

[1] To compare the efficacy of dexmedetomidine and magnesium sulphate in achieving controlled hypotension during FESS.

[2] To assess hemodynamic stability, quality of the surgical field and surgeon satisfaction.

[3] To evaluate the requirement for rescue analgesia and sedation scores postoperatively.

[4] To record and compare adverse effects associated with both agents.

## Materials and Methods:

This hospital-based observational study was conducted at the Department of Anaesthesiology, Gandhi Medical College and associated Hamidia Hospital, Bhopal, from August 2022 to December 2023, following Institutional Ethics Committee approval. A total of 60 ASA grade I and II patients, aged 18 to 60 years, scheduled for elective FESS under general anaesthesia, were enrolled and randomly assigned to two equal groups of 30 each: Group D (dexmedetomidine) and Group M (magnesium sulphate). Patients with cardiovascular or neurological comorbidities, seizure disorders, ASA grade III or higher, pregnancy, or those who refused consent were excluded from the study. Preoperative evaluation included a detailed medical history and routine investigations such as complete blood count, urine analysis, renal and liver function tests, coagulation profile, electrolytes, ECG and chest X-ray for patients above 40 years. Airway assessment was performed using the Mallampati grading and written informed consent was obtained from all participants. On the day of surgery, baseline vitals, including heart rate (HR), systolic blood pressure (SBP), diastolic blood pressure (DBP), mean arterial pressure (MAP) and oxygen saturation (SpO2), were recorded. Patients were premedicated with intravenous glycopyrrolate, fentanyl and midazolam. General anaesthesia was induced using intravenous propofol and succinylcholine, followed by endotracheal intubation and maintenance on controlled ventilation with sevoflurane. Intravenous paracetamol (15 mg/kg) was administered for intraoperative analgesia. Patients in Group D received an intravenous loading dose of dexmedetomidine (1 mcg/kg) over 10 minutes, followed by a continuous infusion of 0.5-1 mcg/kg/hr. Patients in Group M received an intravenous loading dose of magnesium sulphate (40 mg/kg) over 10 minutes, followed by a continuous infusion of 10-15 mg/kg/hr. Throughout the procedure, heart rate, blood pressure and oxygen saturation were monitored at regular intervals. The primary outcome was the ability of each agent to maintain controlled hypotension, defined as a mean arterial pressure (MAP) of 55-65 mmHg. Secondary outcomes included hemodynamic stability, quality of the surgical field (assessed using the Boezaart surgical field grading scale), surgeon satisfaction scores, sedation levels and adverse effects. At the end of the surgery, neuromuscular blockade was reversed using intravenous glycopyrrolate and neostigmine and patients were monitored postoperatively for sedation scores, rescue analgesia requirements and any complications. Data were entered into Microsoft Excel and analyzed using JAMOVI (version 2.6.13). Frequency distributions and cross-tabulations were generated. Pearson's chi-square test assessed relationships between variables. Quantitative data are presented as mean and standard deviation and differences between groups were assessed using independent samples t-tests. Independent samples t-tests were used to compare means for continuous variables, while the chi-square test or Fisher's exact test (where appropriate) were used for categorical variables. Effect sizes were calculated using Cohen's d for t-tests. A p-value < 0.05 was considered statistically significant.

## Results:

The mean ages for both Group D and Group M were quite similar, with Group D having a mean age of 44.3 years and Group M having a mean age of 43.5 years. The overall mean age for the entire cohort was 43.9 years. The statistical analysis using an unpaired t-test confirmed no significant difference in the mean ages between Group D and Group M, with a p-value of 0.774. Approximately 63.3% of males and 36.7% of females were in Group D, while 53.3% of males and 46.7% of females were in Group M. Overall, the gender distribution between the two groups was balanced. The Pearson Chi-Square test showed no significant association between gender and treatment group allocation. With a p-value of 0.432, the test did not reach statistical significance, suggesting that the difference in gender distribution between Group D and Group M was not meaningful. In Group D, 10.0% of the participants were classified as ASA Grade I and 90.0% as Grade II. On the other hand, in Group M, 16.7% were Grade I and 83.3% were Grade II. The relationship between ASA grade and the treatment group was assessed using the Pearson Chi-Square test, which resulted in a p-value of 0.448. This indicates that there was no significant association between the two variables. Therefore, the differences in ASA grade distribution between Group D and Group M were not significant. The mean heart rates for Group D and Group M were compared at different time intervals, including baseline, after loading the drug dose, after induction and during several post-operative periods. Before drug administration, Group D had a mean heart rate of 78.33 ± 4.45 beats per minute (bpm), whereas Group M had a mean heart rate of 77.53 ± 3.58 bpm (Figure 1 see PDF). This initial difference between the two groups was statistically not significant (T = 0.766, p = 0.447), indicating comparable baseline heart rates. After the loading dose of the drug was administered, both groups experienced a reduction in heart rate. Group D showed a mean heart rate of 77.53 ± 3.59 bpm and Group M had a mean of 87.13 ± 10.93 bpm (Figure 1 see PDF). This reduction was statistically significant (T = -4.56, p < 0.001), indicating that both drugs effectively decreased heart rate compared to baseline. Following induction, the difference in mean heart rate between the two groups persisted, with Group D at 75.27 ± 7.49 bpm and Group M at 82.47 ± 13.78 bpm (Figure 1 see PDF). This difference was statistically significant (T = -2.51, p = 0.014), indicating that Group D maintained a lower heart rate compared to Group M even after induction (Figure 1 see PDF). Throughout the surgical procedure, differences in mean heart rate between the two groups continued to be statistically significant at various time intervals, including at 15 minutes (p = 0.007) and up to 90 minutes post-induction (p < 0.05), demonstrating sustained lower heart rates in Group D compared to Group M. Even after extubation, Group D maintained a lower mean heart rate (66.7 ± 7.51 bpm) compared to Group M (74.43 ± 10.88 bpm), with the difference being statistically significant (T = -3.20, p = 0.002) (Figure 1 see PDF). The results show that dexmedetomidine effectively reduced heart rate throughout the surgical procedure compared to magnesium sulphate, indicating its potential efficacy in inducing controlled hypotension for FESS. The systolic blood pressure of Group D (dexmedetomidine) and Group M (magnesium sulphate) at different time intervals was also compared. Before drug administration, at the basal level, Group D had a mean systolic blood pressure of 118.17 ± 4.53 mmHg (Table 1 and Figure 2 see PDF), while Group M had a mean of 119.80 ± 2.64 mmHg (Table 1 and Figure 2 see PDF). This initial difference was statistically not significant (T = -1.704, p = 0.94), indicating comparable baseline systolic blood pressure between the groups. After the loading dose of the drug, both groups experienced reductions in systolic blood pressure. Group D showed a mean systolic blood pressure of 115.4 ± 4.64 mmHg and Group M had a mean of 125.2 ± 4.35 mmHg (Table 1 and Figure 2 see PDF). This reduction was statistically significant (T = -8.43, p < 0.001), indicating that both drugs effectively decreased systolic blood pressure compared to baseline. After induction, the difference in mean systolic blood pressure between the two groups persisted, with Group D at 114.6 ± 7.36 mmHg and Group M at 120.07 ± 6.94 mmHg (Table 1 and Figure 2 see PDF). This difference was statistically significant (T = -2.96, p = 0.004), indicating that Group D maintained a lower systolic blood pressure compared to Group M even after induction. Throughout the surgery, differences in mean systolic blood pressure between the groups were statistically significant at various time intervals, particularly at 15 minutes (p = 0.001) and up to 65 minutes post-induction (p < 0.05), indicating sustained lower systolic blood pressure in Group D compared to Group M. After extubation, Group D maintained a lower mean systolic blood pressure (109.13 ± 7.89 mmHg) compared to Group M (110.67 ± 8.43 mmHg), but the difference was not statistically significant (T = -0.72, p = 0.469) (Table 1 and Figure 2 see PDF). These results indicate that dexmedetomidine effectively reduced systolic blood pressure throughout the surgical procedure compared to magnesium sulphate, supporting its potential efficacy in inducing controlled hypotension for FESS. This trend was also observed in diastolic blood pressure and mean arterial pressure, confirming the hemodynamic advantages of dexmedetomidine over magnesium sulphate.

## Discussion:

This study compared dexmedetomidine and magnesium sulphate for controlled hypotension during FESS, evaluating their effects on hemodynamics, surgical field quality and postoperative outcomes. Controlled hypotension is a valuable technique in FESS, aiming to minimize bleeding and improve surgical visualization. Dexmedetomidine, a selective α2-adrenergic agonist, offers sedative, analgesic and sympatholytic properties, contributing to stable hemodynamic and smooth recovery. Magnesium sulphate, known for its vasodilatory and neuro-protective effects, influences trans-membrane ion exchange and inhibits norepinephrine release [9]. The demographic profiles of the two groups were comparable. No statistically significant differences were observed in mean age, ASA grade distribution, or gender distribution between the dexmedetomidine and magnesium sulphate groups. This balanced distribution suggests that any observed differences in outcomes are likely attributable to the study drugs rather than demographic variations. Our findings demonstrate a statistically significant difference in heart rate between the groups after the loading dose, post-induction and at various intraoperative time points (p<0.05). The dexmedetomidine group consistently exhibited lower heart rates throughout the surgical procedure, consistent with its known sympatholytic effects. This aligns with the findings of Bajwa *et al.* who also reported lower heart rates and blood pressure with dexmedetomidine compared to esmolol [4]. However, Ghodraty *et al.* reported similar hemodynamic properties between magnesium sulphate and remifentanil [10]. Similar to heart rate, both systolic, diastolic and mean arterial blood pressure (MAP) showed statistically significant differences between the groups after the loading dose, post-induction and at various intraoperative time points (p<0.05). Dexmedetomidine effectively reduced blood pressure compared to magnesium sulphate. These findings are consistent with studies by Rahman *et al.* which investigated different doses of dexmedetomidine for controlled hypotension [6].

Bayram *et al.* also reported significantly decreased MAP in the dexmedetomidine group, except at the initial stage [3]. Sujay *et al.* (2021) further supported the superiority of dexmedetomidine over labetalol for controlled hypotension and surgical field visibility during FESS [11]. Our study found statistically significant differences in MAP between the groups after the loading dose, post-induction and at various intraoperative time points up to 65 minutes (p<0.05). Dexmedetomidine consistently maintained lower MAP values. While MAP reduction is crucial for minimizing bleeding, it's important to balance this with adequate organ perfusion. Khalifa *et al.* successfully achieved a target MAP of 55-65 mmHg using various hypotensive agents, including dexmedetomidine and magnesium sulphate [12]. Sieskiewicz *et al.* highlighted the importance of maintaining a heart rate of around 60 bpm to optimize the surgical field without excessively lowering MAP [13]. No statistically significant difference was observed between the groups regarding the need for rescue analgesia. This suggests that both dexmedetomidine and magnesium sulphate provide comparable intraoperative analgesia. Our findings are in line with Ossama *et al.* [14] study, which reported no difference in postoperative pain between dexmedetomidine and magnesium sulphate groups. Dong *et al.* [15] discovered that the drug decreased the requirement of opioids as well as the satisfaction of the control of pain in the entire postoperative period. Yu *et al.* [16] discovered that intravenous application of magnesium sulphate might decrease the consumption of postoperative analgesia as well as postoperative pain. The mean Aldrete scores were similar between the two groups, indicating comparable postoperative recovery profiles. This suggests that both agents have similar effects on recovery from and surgery. Lee *et al.* reported higher sedation levels and longer Aldrete score duration with dexmedetomidine compared to remifentanil [17]. Ozcan *et al.* also observed slower recovery with compared to remifentanil [18]. Surgeon satisfaction, assessed using a Likert scale, was significantly higher in the dexmedetomidine group (p<0.001). This suggests that dexmedetomidine provides superior surgical field visualization compared to magnesium sulphate. This finding is consistent with the study by Faranak *et al.* which reported lower bleeding scores and better surgeon satisfaction in the dexmedetomidine group. Sedation scores were similar between the two groups at all measured time points, indicating that both drugs provided comparable levels of sedation during FESS. This aligns with the findings of Ozcan *et al.* who concluded that dexmedetomidine and remifentanil have similar sedation effects during FESS [18]. Hypotension and bradycardia were observed in four patients in the dexmedetomidine group, requiring intervention with crystalloids, ephedrine and atropine. Nausea and vomiting occurred in two patients in the magnesium sulphate group despite routine antiemetic prophylaxis. These findings are consistent with previous reports. Bayram *et al.* also reported bradycardia in both dexmedetomidine and magnesium sulphate groups [3]. Nayantara *et al.* [19] and Chhabra *et al.* [20] conducted a similar study and concluded that the target MAP of 60-70 mmHg or a 30% reduction from the baseline MAP was achieved significantly earlier in group D as compared with group M. Meenakshi *et al.* [21] and Catherine Kapoor *et al.* [22] conducted a similar study and observed a decrease in MAP which is more in dexmedetomidine group than magnesium sulphate group. These results are concomitant with our study.

## Conclusion:

Dexmedetomidine proved superior to magnesium sulphate in achieving and maintaining controlled hypotension. This reflected consistently in lowering mean arterial pressure (MAP) values with greater hemodynamic stability, particularly in heart rate control. It also resulted in a clearer surgical field, leading to increased surgeon satisfaction. While both agents demonstrated acceptable safety profiles, dexmedetomidine was associated with fewer instances of postoperative nausea and vomiting. Individualized patient assessment and meticulous hemodynamic monitoring remain crucial for safe and effective controlled hypotension, regardless of the chosen agent.

## Limitations of the study:

Quantitative assessment of blood loss could not be performed. The anaesthesiologist administering anaesthesia was blinded to the study drug. Invasive blood pressure monitoring was not available. The sample size of 60 patients may limit the generalizability of the findings. Additionally, the inclusion criteria restricted the study to ASA grade I and II patients, which may not reflect outcomes in patients with higher ASA grades.

## Figures and Tables

**Table 1:** Comparison of Systolic blood pressure between the groups

**Time Intervals**	**Group D [Mean ± SD]**	**Group M [Mean ± SD]**	**T value**	**P value**
Basal	118.17±4.53	119.80±2.64	-1.70, df=58	0.094
After loading the dose of drug	115.4±4.64	125.2±4.35	-8.43, df=58	<0.001
After Induction	114.6±7.36	120.07±6.94	-2.96, df=58	0.004
At 15 minutes	105.67±8.19	112.13±6.83	-3.32, df=58	0.001
At 25 minutes	103.67±7.22	107.73±7.71	-2.10, df=58	0.039
At 35 minutes	102.5±6.85	105.2±8.36	-1.36, df=58	0.176
At 45 minutes	102.93±6.6	105±7.17	-1.16, df=58	0.249
At 55 minutes	103.07±5.98	104.47±7.51	-0.79, df=58	0.427
To 65 minutes	112.03±7.63	116.83±10.57	-2.01, df=58	0.048
To 75 minutes	106.6±7.2	109.1±8.73	-1.20, df=58	0.231
At 85 minutes	106.4±7.64	108.67±8.44	-1.09, df=58	0.279
At 90 minutes	106.97±5.5	109.67±7.48	-1.59, df=58	0.116
After Extubation	109.13±7.89	110.67±8.43	-0.72, df=58	0.469
Unpaired 't' test applied.
P value <0.05 was considered
statistically significant.
